# An actionable anti-racism plan for geoscience organizations

**DOI:** 10.1038/s41467-021-23936-w

**Published:** 2021-06-22

**Authors:** Hendratta N. Ali, Sarah L. Sheffield, Jennifer E. Bauer, Rocío P. Caballero-Gill, Nicole M. Gasparini, Julie Libarkin, Kalynda K. Gonzales, Jane Willenbring, Erika Amir-Lin, Julia Cisneros, Dipa Desai, Maitri Erwin, Elisabeth Gallant, Kiara Jeannelle Gomez, Benjamin A. Keisling, Robert Mahon, Erika Marín-Spiotta, Leiaka Welcome, Blair Schneider

**Affiliations:** 1 Fort Hays State University, Hays, KS USA; 2 University of South Florida, Tampa, FL USA; 3 University of Michigan, Ann Arbor, MI USA; 4 GeoLatinas, George Mason University, Brown University, Fairfax, VA, USA; 5 Tulane University, New Orleans, LA USA; 6 Michigan State University, East Lansing, MI USA; 7 National Institute of Health, Bethesda, MD USA; 8 Stanford University, Stanford, CA USA; 9 American Water Works Association, Denver, CO USA; 10 University of Illinois Urbana Champaign, Champaign, IL USA; 11 University of Massachusetts Amherst, Amherst, MA USA; 12 Microsoft, Redmond, WA USA; 13 University of Cambridge, Cambridge, UK; 14 University of Texas at Austin, Austin, TX, USA; 15 Columbia University, New York, NY USA; 16 The University of New Orleans, Orleans, LA USA; 17 University of Wisconsin-Madison, Madison, WI USA; 18 Colorado School of Mines, Golden, CO, USA; 19 University of Kansas, Lawrence, KS, USA

**Keywords:** Science in culture, Policy

## Abstract

Geoscience organizations shape the discipline. They influence attitudes and expectations, set standards, and provide benefits to their members. Today, racism and discrimination limit the participation of, and promote hostility towards, members of minoritized groups within these critical geoscience spaces. This is particularly harmful for Black, Indigenous, and other people of color in geoscience and is further exacerbated along other axes of marginalization, including disability status and gender identity. Here we present a twenty-point anti-racism plan that organizations can implement to build an inclusive, equitable and accessible geoscience community. Enacting it will combat racism, discrimination, and the harassment of all members.

## Abstract (web summary)

Racism thrives in geoscience. We present an antiracism plan to support the recruitment, retention and success of Black, Indigenous, and other people of color in geoscience. Our action plan can be adapted by any organization to remove barriers to participation for all marginalized geoscientists.

## Background

Racism thrives in geoscience^1^. Geoscience organizations function alongside the same racist ideologies and practices shaping society. In North America, the historical legacy of racism—for example: the enslavement of Black people, forced migration of Indigenous peoples, the internment of Japanese Americans, and detainment of Latinx, immigrant children—is intertwined with our systems of power. The imbalance of power dictates who has access to resources like inherited wealth, clean water, adequate nutrition, healthcare, effective education, and who is policed, imprisoned, and killed. Many people around the world become confronted with these realities of racial power dynamics only when they see graphic recordings of people of color (POC) who are murdered, discriminated against, or harassed in viral internet videos. Summer 2020 became a unique key moment of reckoning when several viral videos of the harassment and murder of Black individuals in the US ignited global protests decrying racism.

Racism has led to the geosciences becoming one of the least diverse among all science and engineering fields^2^. Thus, as is often the case following a national tragedy, numerous organizations—professional societies, colleges, departments, industries, labs, government agencies, and non-profits that house the geoscience community—released statements calling out societal racism and discrimination that unavoidably permeates into geoscience culture. However, these statements often fail to account for the sustained historical efforts, made by Black and other minoritized geoscientists to diversify the discipline and whose efforts in many instances have been forgotten, ignored, and erased^3^. We assert that these statements of support, though important first steps, are generally ineffective at assisting minoritized people (e.g., Black, Indigenous and other People of Color (BIPOC), disabled people, lesbian, gay, bisexual, transgender, queer, and genderqueer (LGBTQ+) people, foreign nationals, and/or women) in fighting racism or discrimination. Certainly, the significant lack of diversity in the geosciences^1,4–6^ cannot be addressed without effective actions that first address racism and its effects on access, inclusion, equity, and justice. While the geosciences have unique structures that may exacerbate racism and the exclusion of minoritized communities (e.g., in field-based education, and access to remote location fieldwork), the geosciences are not unique, as a discipline, in the inherent racism within its systems. Thus we believe this plan is also applicable to other disciplines. Similarly, in addition to focusing on anti-Black racism, geoscience organizations must also consider and engage with how other historically underrepresented, marginalized, and other POC groups have been excluded from the discipline, and then take a more proactive approach to inclusion.

While many people understand and acknowledge that racism exists within society, it can be more difficult to see the racism that is manifesting within spaces held dear. This is certainly true for scientific organizations, considered bastions of logic, separate from humanistic concerns. Yet, geoscientists cannot continue to be complicit in racism, discrimination, and inaction^1^. As Dr. Angela Y. Davis has said, “In a racist society, it is not enough to be non-racist, we must be anti-racist”^7^—and anti-racism requires action.

## Essential constructs for effective anti-racism

**Fig. 1:**
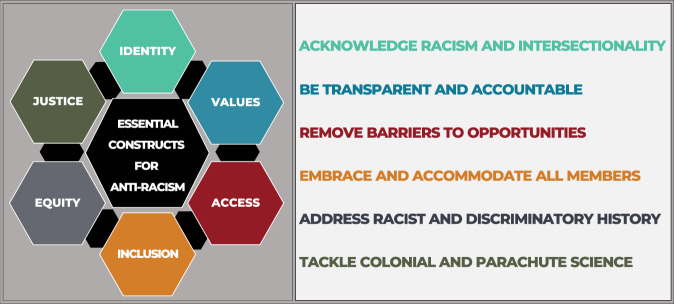
Six essential constructs for effective anti-racism.

**Fig. 2:**
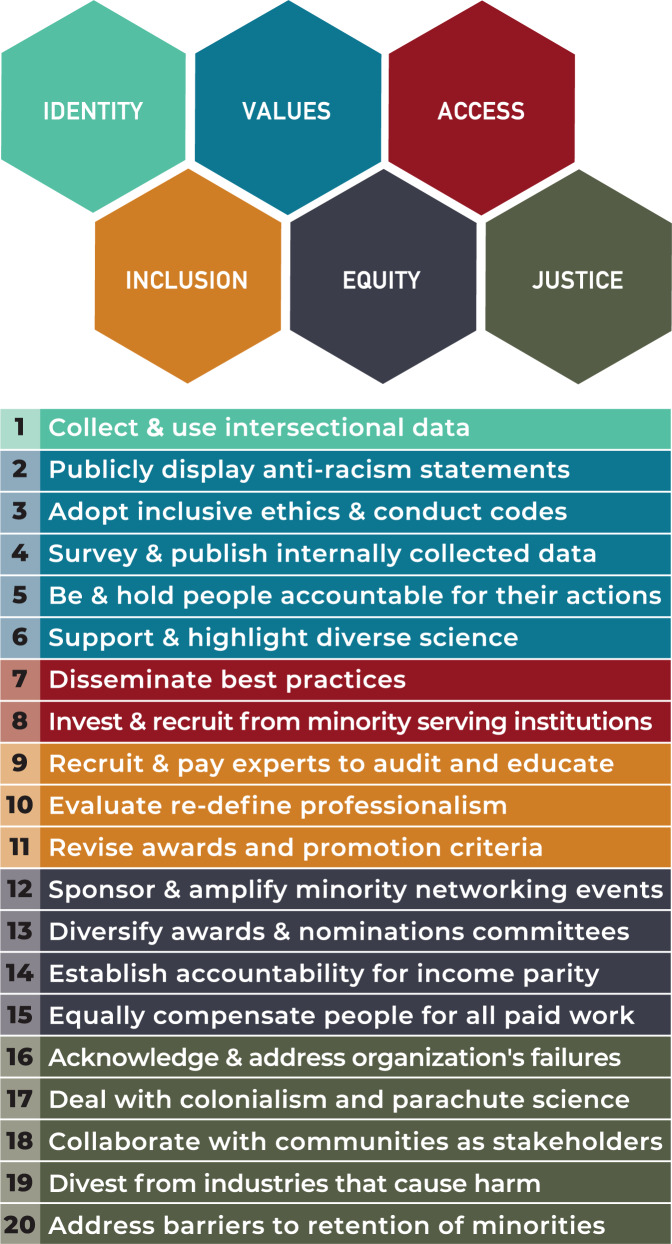
Twenty action steps to build a robust anti-racist organization. Each action step is associated to an essential construct.

For an organization to be anti-racist and equitable, it needs to ask and answer some difficult yet important questions: Who is in the organization? Who benefits from the status quo? Who holds power, and who feels safe? Who is left out, who is powerless, and who feels unsafe? And ultimately, Why? Why do these differences exist? In considering these questions, this group—consisting of BIPOC, white, LGBTQ+, straight, disabled, abled, immigrant, non-immigrant, women, men, and genderqueer individuals—identifies 20 concrete actions that organizations must take to become anti-racist. These 20 actions are organized around six constructs—*identity*, *values*, *access*, *inclusion*, *equity*, and *justice*—vital for anti-racist thinking (Figs. 1 and 2).

### Identity

In considering anti-racism in the geosciences, we cannot ignore the intersecting identities of marginalized people^8^. We must acknowledge the added burden of inequalities and oppression experienced by people and communities with these intersectional identities, such as Black women who are subjected to both sexism and racism, or when class status, disability, gender expression, or sexual identity intersect with other minoritized identities^9–12^. Indeed, focusing on only one axis of diversity has led to some gains only in more white women working as geoscientists, but has not increased the participation of BIPOC and other marginalized peoples^5^.

While a singular approach to anti-discrimination cannot work for all groups, explicitly anti-racist policies will lift all minoritized identities in a ripple effect. For example, re-evaluating fieldwork policies and practices to ensure safety for BIPOC geoscientists will also relieve barriers placed on disabled, lower income, and/or LGBTQ+ people^8,11,13–17^. Similarly, the presence of BIPOC in spaces expands opportunities for students to identify with allies and mentors, with similar lived experiences. Community is important because, if not adequately prepared for the role, white mentors or other mentors with dissimilar identities from those of the mentee, frankly, may do more harm than good for the BIPOC or other minoritized students, and may further reinforce an unjust and racialized hierarchy instead of providing meaningful mentoring for the minoritized students^18^. White mentors or mentors from a majority group should be adequately prepared to mentor individuals from marginalized and underrepresented groups.

Achieving anti-racism requires two-way communication, trust-building, active listening, and believing the experiences of BIPOC. Before all else, organizations must collect data on the experiences of historically minoritized and excluded social groups, and use these data to engage in action-oriented conversations (ACTION #1)^19,20^ in order to understand their experiences within the discipline and organizations. Proactive communication is vital, lest emergent events force organizations into unplanned actions without a clear direction, without adequate training and resources, or without an informed leadership.

### Values

Many organizations have publicly declared themselves to be anti-racist. This articulation of *values* must be done with complete *transparency* and executed with *accountability*. First, organizations should ensure that their anti-racism and anti-discrimination statements, including anticipated actions and expected outcomes, are shared publicly and accessibly (ACTION #2). Second, anti-racism and anti-discrimination language should be explicitly adopted into codes of ethics and codes of conduct (ACTION #3). Third, organizations should strive for transparency by openly seeking information, feedback, and acknowledging their successes, failures, and opportunities for improvement. This can occur through sharing of, and soliciting feedback from published annual, data-rich reports of the self-reported, intersectional demographics of their members, awardees, and leaders (ACTION #4), because as scientists, we understand and value data, and must therefore use data to measure progress and ensure data-driven accountability.

Organizations must also develop policies and procedures to show how they will hold the institution and individuals accountable for their actions or their inactions (ACTION #5). Geoscience societies in particular can provide leadership for the community’s development of policies. The important role of societies for policy setting is, for example, evident in the role that the American Geophysical Union^19^, and the Paleontological Society^20^ played in leading the geoscience’s adoption of stronger anti-harassment policies, thus providing a model for enacting change within other organizations.

As a clear reflection of values, organizations should actively work towards parity in representation by focusing on increasing diversity as an integral part of science (ACTION #6). For example, speaker series—from department seminars to conference keynotes—should regularly identify and feature minoritized people who represent the technical and stakeholder communities. In addition, organizations should require third party audits to assess diversity, equity, and inclusion (DEI) performance in their hiring, promotion, and related constructs as well as audits that show how they value research and scholarship that is meaningful to diverse communities, for example, community-inspired research that can build trust and broaden participation of Native Americans^21^. These audits should also have an impact on their potential for future funding, program accreditations, their ranking, and prestige.

### Access

Access implies that individuals can obtain the resources they need to safely pursue their science endeavors; regardless of location, instrumentation, site accessibility, and their identity. Historically, access has been limited to mostly able-bodied, white, cisgender, heterosexual men. As the geosciences strive to be more accessible, the community must recognize that BIPOC and other marginalized geoscientists are not always safe in geoscience spaces. For example, holding objects (e.g., a rock hammer) has been viewed as “suspicious” and, continues to be, used as a reason to call the police on Black people, which can lead to the death of Black individuals, entirely because of racial profiling and an unjustified fear of Black people^17,22^. Organizations can lead by requiring and disseminating best practices that make all programs safe for, and accessible to, everyone (ACTION #7). This requirement includes incorporating anti-racism into all spaces where geoscience happens^23^—in the field, in laboratories, virtually, at events and in classrooms—by encouraging the reevaluation of training requirements for learners and aspiring geoscientists, and invest in spaces like HBCUs (Historically Black Colleges and Universities)^24^, TCUs (Tribal Colleges and Universities), and HSIs (Hispanic Serving Institutions)^25^, that serve BIPOC and other marginalized geoscientists (ACTION #8). In this effort, scientific societies, DEI non-profit organizations, and funding agencies can individually, or in partnerships, leverage their influence to incentivize, encourage, and induce academic institutions, departments, research labs, and field stations and camps within their disciplines to adopt norms and practices that foster inclusion, collaboration with, and the safety of minoritized individuals. For example, collaborative partnerships like the National Science Foundation-funded ADVANCEGeo Partnerships (https://serc.carleton.edu/advancegeo/), between the Earth Science Women’s Network, Association for Women Geoscientists, and the American Geophysical Union, empowers scientists at societies and institutions to transform their workplace climate through tailored trainings, workshops, and expected outcome assessments.

### Inclusion

Inclusion must start with acknowledging that individuals have multiple identities that intersect within a matrix of domination and oppression^26,27^. Organizations should actively work to understand, and acknowledge the lived experiences of BIPOC and other minoritized people, then assess how racism and discrimination have impacted individual’s ability to thrive, succeed, and belong^8,28^. This necessitates hiring BIPOC and other marginalized experts to offer recurring trainings to educate and train leaders, staff, and members to identify and eliminate the structural and implicit biases within their geoscience community (ACTION #9).

The community should acknowledge that most geoscience organizations were built by, and for, white people at a time when many forms of discrimination were legal and that these organizations have continued, for the most part, to uphold customs and expectations that have racist impacts. For example, current expectations around manners, clothing, hair, professional attire, language, and diction are all racist at their core. Thus, organizations should recognize how current “professional” expectations negatively impact BIPOC members^29,30^, and make appropriate changes (ACTION #10). Explicit policies and procedures require similar examination. For example, lengthy nomination procedures for awards and leadership positions in societies put undue burden on minoritized people who often do not have the required network connections within the larger white community and are less likely to be sponsored by senior (often white) male scientists^31,32^. A review and revision of the criteria for honors and awards, promotion, and leadership selection are critical to secure inclusion (ACTION #11).

### Equity

To ensure equity, organizations must intentionally recruit, provide opportunities for, and enact practices to retain BIPOC and other marginalized people^33^. Therefore, in addition to supporting specific minority targeted initiatives and programs^5^, organizations should directly sponsor inclusion networking events for BIPOC geoscientists at all their large gatherings such as recruitment, alumni, and conference events (ACTION #12). This action is necessary because, in their current form, large professional gatherings are still very exclusive of minoritized individuals because of challenges due to cost, accessibility, belonging, and representation. As a result of low representation at these gatherings, the contributions of BIPOC scholars who do attend are often invalidated and devalued, further enabling anti-BIPOC racism and discrimination. Therefore, organizations should purposefully work to remove barriers that prevent minoritized geoscientists, who attend and participate, from progressing to leadership positions. To achieve this, organizations must actively revise their recruiting and selection criteria, to diversify their candidate pool, and their awards and selection committees^34,35^ (ACTION #13). Further, pay inequity exists. This inequity is a historical and ongoing legacy of discrimination that contributes to the financial hardship, which is a reality for many minoritized geoscientists. Organizations can address pay inequities by actively advocating for, and creating accountability measures to check income parity (ACTION #14). These measures should also include equally compensating BIPOC and other marginalized geoscientists for all paid organizational duties (ACTION #15).

### Justice

Anti-racism must acknowledge the historical wrongdoings of colonialism and racism, and work to redress and create a just community. To start, each geoscience organization should identify and acknowledge ways the organization has failed its BIPOC and other marginalized members, both structurally and individually (ACTION #16), followed by taking specific actions to address these failures. This is especially salient in local and regional organizations within which the diverse experiences of members can be leveraged to create change in the local communities.

Second, geoscience organizations must acknowledge and address the impact of the historical and ongoing erasure of Indigenous knowledge enacted through colonialist practices (ACTION #17). Colonialism negatively impacts—including death and loss of land—BIPOC in North America and around the world^36^. Colonialism empowers settlers who extract cultural and natural resources to, for example, populate museums and private collections, disrespecting autonomous rights^37,38^. Furthermore, modern-day geoscience is still pervaded by a research model (e.g., “parachute science”^39,40^) that exploits and extracts knowledge from remote locations, with little to no credit for, or participation by the Indigenous or local communities, thus limiting the science to a narrow band of questions and solutions dictated, and often published exclusively for, and by the white majority.

Organizations must also acknowledge the environmental injustice caused by geoscience endeavors. For example, BIPOC are more negatively affected by environmental hazards, have limited access to resources, and disproportionately experience the negative impacts of a changing environment^41,42^. Therefore, BIPOC communities must be included as engaged stakeholders to participate in any environmental action, research, and resource extraction happening within their spaces (ACTION #18). In addition, acknowledging that the natural resources that help support our organizations often come from global extractive industries whose activities disproportionately and negatively impact BIPOC communities, organizations must consider how to divest from, demand remediation and reparations from industries and institutions that have harmed people and the environment (ACTION #19). Additionally, although extractive industries attract BIPOC individuals at a significantly higher rate into the geosciences, organizations should also recognize, acknowledge, and strive to resolve the similarly higher rates of attrition for these same BIPOC individuals (ACTION #20). Thus, minoritized individuals need mentors, sponsors, and advocates to explicitly facilitate equity in networks, within the industries, institutions, and organizations.

## Conclusion

Enacting this anti-racism plan requires planning. Organizations should select leaders from diverse backgrounds to identify specific anti-racist actions from this plan, and support them to enact those actions; this recommendation aligns with the Unlearning Racism in the Geosciences program (https://urgeoscience.org/) that launched in early 2021. Developing specific strategies from this plan that will lead to a more anti-racist organization will depend on the organization’s—Belonging, Accessibility, Justice, Equity, Diversity and Inclusion (BeAJEDI)—standing, its unique needs, leadership vision, and actions selected. Some steps in the plan are comparatively easy to implement while others require more resources, planning, and stakeholder engagement, and would require bold organizational leadership and strategic scaffolding to enact. As of the writing of this article, many geoscience organizations have acted on some of the steps in this plan. However, most have not advanced past undertaking only the few, simple steps that lead to instantly visible, short-term results (e.g., creating new a DEI task force, a new diversity award for minoritized individuals, or a scholarship for students from minoritized communities). These actions are quick fixes that can initiate conversation and also reward professionally deserving individuals. Long term, however, these quick fixes do not alter structural racism. By systematically revisiting this anti-racism action plan, and by building on the steps initiated or already taken, organizations can incrementally continue to move towards becoming fundamentally anti-racist. The next few years will be crucial in revealing how the geoscience community and other STEM disciplines tackle the presence of racism and discrimination in their communities. This plan is a practical roadmap. Where will it take your geoscience organization?
